# A genome-wide association study of antidepressant response in Koreans

**DOI:** 10.1038/tp.2015.127

**Published:** 2015-09-08

**Authors:** W Myung, J Kim, S-W Lim, S Shim, H-H Won, Seonwoo Kim, Sangha Kim, M-S Lee, H S Chang, J-W Kim, B J Carroll, D K Kim

**Affiliations:** 1 Department of Psychiatry, Samsung Medical Center, Seoul, Korea; 2 Sungkyunkwan University School of Medicine, Seoul, Korea; 3 Center for Clinical Research, Samsung Biomedical Research Institute, Seoul, Korea; 4 Biostatistics Team, Samsung Biomedical Research Institute, Seoul, Korea; 5 Department of Psychiatry, College of Medicine, Korea University, Seoul, Korea; 6 Soonchunhyang Medical Institute, College of Medicine, Soonchunhyang University, Asan, Korea; 7 Department of Laboratory Medicine and Genetics, Samsung Medical Center, Seoul, Korea; 8 Pacific Behavioral Research Foundation, Carmel, CA, USA

## Abstract

We conducted a three-stage genome-wide association study (GWAS) of response to antidepressant drugs in an ethnically homogeneous sample of Korean patients in untreated episodes of nonpsychotic unipolar depression, mostly of mature onset. Strict quality control was maintained in case selection, diagnosis, verification of adherence and outcome assessments. Analyzed cases completed 6 weeks of treatment with adequate plasma drug concentrations. The overall successful completion rate was 85.5%. Four candidate single-nucleotide polymorphisms (SNPs) on three chromosomes were identified by genome-wide search in the discovery sample of 481 patients who received one of four allowed selective serotonin reuptake inhibitor (SSRI) antidepressant drugs (Stage 1). In a focused replication study of 230 SSRI-treated patients, two of these four SNP candidates were confirmed (Stage 2). Analysis of the Stage 1 and Stage 2 samples combined (*n*=711) revealed GWAS significance (*P*=1.60 × 10^-8^) for these two SNP candidates, which were in perfect linkage disequilibrium. These two significant SNPs were confirmed also in a focused cross-replication study of 159 patients treated with the non-SSRI antidepressant drug mirtazapine (Stage 3). Analysis of the Stage 1, Stage 2 and Stage 3 samples combined (*n*=870) also revealed GWAS significance for these two SNPs, which was sustained after controlling for gender, age, number of previous episodes, age at onset and baseline severity (*P*=3.57 × 10^-8^). For each SNP, the response rate decreased (odds ratio=0.31, 95% confidence interval: 0.20–0.47) as a function of the number of minor alleles (non-response alleles). The two SNPs significantly associated with antidepressant response are rs7785360 and rs12698828 of the *AUTS2* gene, located on chromosome 7 in 7q11.22. This gene has multiple known linkages to human psychological functions and neurobehavioral disorders. Rigorous replication efforts in other ethnic populations are recommended.

## Introduction

Response rates in drug treatment of major depression are unsatisfactory.^1^ Initial antidepressant treatments fail in at least one-third of patients.^2^ In addition, there are no biomarkers of treatment response. On the basis of family studies of response to antidepressants,^3, 4^ genetic markers hold promise for improving this record.^5^

Selecting candidate genes related to antidepressant response is difficult because of our limited knowledge of the underlying biology. In addition, most pharmacogenetic studies that have focused on a few candidate genes (for example, serotonin transporter gene, *SLC6A4*) have shown results that lack sufficient predictive power to be useful in clinical practice.^6^ Recently, several genome-wide pharmacogenetic studies of antidepressant treatment in major depressive disorder (MDD) were conducted to overcome these limitations.^7, 8, 9, 10, 11^ However, none of their full-sample results reached genome-wide levels of statistical significance, and the top results were also inconsistent.^6^ These failures underscore the heterogeneous phenotype and the complex nature of clinical depression. In addition, these large, multisite studies risk being confounded by heterogeneity of case material, ethnicity and recruitment practices.^12, 13^

In this study, we conducted a genome-wide pharmacogenetic study in ethnically homogeneous patients, with careful quality control. The discovery phase was conducted in a single, experienced clinical research site. The resulting significant findings were then validated in an independent, ethnically identical sample (replication sample). Our hypothesis is that common DNA variations are associated with antidepressant response to selective serotonin reuptake inhibitors (SSRIs). As a secondary question, we investigated whether the same genomic associations held true for a non-SSRI antidepressant drug in the same homogeneous ethnic group (cross-replication sample).

## Materials and methods

### Definition of cohorts

**Figure 1:**
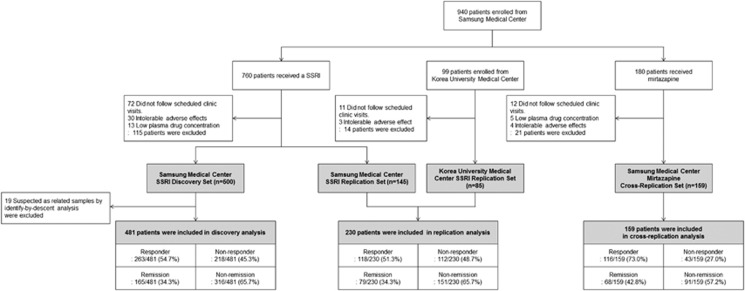
Enrollment, attrition, drug treatments and outcomes of patients in all samples. SSRI, selective serotonin reuptake inhibitor.

We enrolled a total of 1039 patients overall. For the discovery and replication phases, we enrolled 859 SSRI-treated patients. Of these, 760 were seen at the Samsung Medical Center (SMC; Seoul, Korea), and 99 were seen contemporaneously at a second site—Korea University Medical Center (Seoul, Korea). After dropouts as detailed in Figure 1, there were 500 completer patients in the SSRI discovery set in the first phase of this project and 230 completer patients in the SSRI replication set in the later phase of the project. Thus, the overall protocol completion rate was 85.0% (730/859) among the SSRI-treated patients. In addition, 19 completer cases were removed from analysis of the discovery set because identity-by-descent analysis^14^ revealed possible relatedness with a previously enrolled patient. The enrolled cross-replication set comprised 180 mirtazapine-treated patients, all of whom were seen at SMC (Figure 1). Of these, 159 completed the protocol with a protocol completion rate of 88.3% (dropouts detailed in Figure 1). Thus, the overall successful protocol completion rate was 85.5% (889 of 1039 enrolled cases). The completer cohorts analyzed after removal of the identity-by-descent cases comprised 481 patients in the SSRI discovery set; 230 in the SSRI replication set; and 159 in the mirtazapine cross-replication set—a total of 870 cases (Figure 1 and Table 1). The SSRI drugs given in the 481-patient discovery set and in the 230-patient replication set are shown in Table 1.

### Study patients from the SMC

We enrolled the patients from April 1999 through April 2012. A total of 940 patients with MDD, were recruited from the clinical trials program of the Samsung Medical Center Geropsychiatry and Affective Disorder Clinics. These included 101 of 136 subjects reported previously^15^ and recruited within the time window of the present report. All cases were clinically referred and all were of unrelated Korean ancestry. Consistent with current genome-wide association study (GWAS) strategy, the study was conducted in a naturalistic clinical setting rather than in a placebo-controlled clinical trial.^7, 8^

Inclusion criteria were 18 years of age or older, the existence of a current unipolar major depressive episode as verified by *Diagnostic and Statistical Manual of Mental Disorders, Fourth Edition – Text Revision* (DSM-IV-TR) criteria for MDD,^15, 16^ and being capable of providing informed consent. The diagnosis was based on an initial clinical interview, followed by a structured research assessment, the Samsung Psychiatric Evaluation Schedule, which includes the Structured Clinical Interview for DSM-IV.^17^ The final diagnosis was made by a board-certified psychiatrist after review of ongoing clinical observations, medical records, past histories and Structured Clinical Interview for DSM-IV interview. The baseline minimum 17-item Hamilton scale for depression (HAM-D)^18^ score required for enrollment was 15. Exclusion criteria were pregnancy, significant medical conditions, abnormal laboratory baseline values, unstable psychiatric features (for example, suicide attempt), histories of alcohol or drug dependence, seizure, neurological illness including significant cognitive impairment, or concomitant DSM-IV Axis I psychiatric disorder. Patients with MDD who met DSM-IV criteria for the specifier ‘*Severe With Psychotic Features'* were excluded because they would normally receive concurrent antipsychotic medication. No patient had received psychotropic medication within the current episode, an average duration of 3–5 months and a minimum duration of 4 weeks (Table 1).

### Procedures

Patients received monotherapy for 6 weeks with an antidepressant drug. As shown in Figure 1 and Table 1, 481 completer patients in the discovery cohort and 230 completer patients in the replication cohort received one of four allowed SSRI drugs (total 711 SSRI-treated patients), whereas 159 patients in the completer cohort of the cross-replication sample received mirtazapine. The clinician's antidepressant choice was naturalistic, taking account of anticipated adverse effects. Dose titration was completed within 2 weeks. Trough plasma samples were drawn at the end of week 6 for plasma drug concentrations. Lorazepam (0.5–1 mg) was allowed at bedtime for insomnia.

Patients were seen by a psychiatrist, who monitored their adverse events by the Udvalg for Kliniske Undersogelser scale^19^ at weeks 0, 1, 2, 4 and 6. HAM-D was administered by a single trained rater every 2 weeks. During the 13 years of this study, five clinical psychologists performed the HAM-D ratings. All raters had received HAM-D training, supervised by two licensed clinical psychologists. They reviewed and rated demonstration tapes of HAM-D ratings.^20^ An intraclass correlation coefficient of 0.91 was obtained for the 17-item total HAM-D score.

The rater and genotyper were blinded to the hypotheses and to drug assignment. HAM-D and genotype data were not disclosed to the psychiatrist, and the rater was blinded to the genotype data. To maintain the blindness, a trained research coordinator managed the data and schedules. At 6 weeks, response was defined according to standard conventions^21^ as ⩾50% decrease in the HAM-D score. Remission was defined as a HAM-D score of less than 8 at 6 weeks.^22^

Figure 1 shows the flow of patients through the study. As this is a discovery project, outcome analyses included only subjects who completed 6 weeks of treatment with adequate drug plasma levels. Detailed description for the flow is provided in Supplementary Material and Methods (Supplementary Text S1).

The analyzed replication set included 85 completer patients of 99 enrolled patients recruited from the Pharmacogenomic Research Center for Psychotropic Drugs of the Department of Psychiatry, Korea University Medical Center. The procedures followed at this site were closely similar to those described for SMC patients. The significant exceptions were that these patients did not provide samples for antidepressant blood level determinations and there was no information on duration of current episode. The detailed protocols were described in previous reports from Korea University Medical Center.^23, 24^ The protocol was approved by the ethics review board of SMC and by the ethics committee of the Korea University Medical Center. Signed informed consent was obtained from all participants.

### Genotyping

We used the Affymetrix Genome-wide Human single-nucleotide polymorphism (SNP) array chip 6.0 for 905,431SNPs for genotyping the discovery set. Four candidate SNPs (*P*<1.00 × 10^−5^) were identified in the discovery set. These were genotyped in the replication and cross-replication sets using the MassARRAY system (Sequenom, San Diego, CA, USA). For a full description, refer to Supplementary Material and Methods (Supplementary Text S1).

### Statistical analysis

For non-normally distributed continuous variables, results are presented as the median and interquartile range, and nonparametric tests (the Wilcoxon rank-sum test and the Kruskal–Wallis test) were used to compare groups. Categorical variables are summarized as frequencies and proportions. Fisher's exact test was used to compare groups on categorical variables. *P*-values were corrected by Bonferroni's correction in case of multiple testing, and marked as 'corrected *P*'.

The Cochran–Armitage trend test with one degree of freedom was used for testing the association between number of minor alleles and response.^25^ For the replication set, we conducted a focused analysis, selecting candidate SNPs with *P*-values less than 1.0 × 10^−5^ from the discovery set. In the replication and cross-replication analyses, SNPs with *P*-values after Bonferroni's correction less than 0.05 were considered significant. Then, we performed combined analyses with the discovery and replication sets. In this combined analysis, a genome-wide significance threshold (5.0 × 10^−8^) was used.^26, 27^ In addition, we employed multiple logistic regression analysis for each significant SNP in order to examine the association between the number of minor alleles and response with adjustment for possible confounding variables. All statistical analyses were performed using PLINK Version 1.07 (ref. 28) and PASW Statistics, version 18.0 (SPSS, Chicago, IL, USA).

## Results

### Subject characteristics

Clinical and demographic characteristics are shown in Table 1. Subjects were mostly elderly, and experiencing their second or later episode of MDD. Approximately one-fifth of the patients had a positive family history of depression. The median pretreatment HAM-D score was 19, which indicates moderately severe depression. Observed response rates were above 50% in the discovery and replication sets, and a higher response rate was seen in the cross-replication set. The remission rate as a proportion of the response rate was 0.63 in the discovery set, 0.67 in the replication set and 0.59 in the cross-replication set. These proportions are typical of antidepressant trials^29^ and they suggest that rating bias was not present. There were statistically significant but clinically minor differences among sets with respect to gender, age, age at first episode and number of past episodes. Number of episodes, duration of current episode and baseline HAM-D score in the discovery set, gender, duration of current episode and baseline HAM-D score in the replication set, and number of episodes in the cross-replication set were associated with response. Drug choice within the SSRI class differed between the discovery set and the replication set, but was not associated with response rate differences across the two sets (Table 1).

### Genetic-association analysis of SSRI treatment response in discovery set

**Figure 2:**
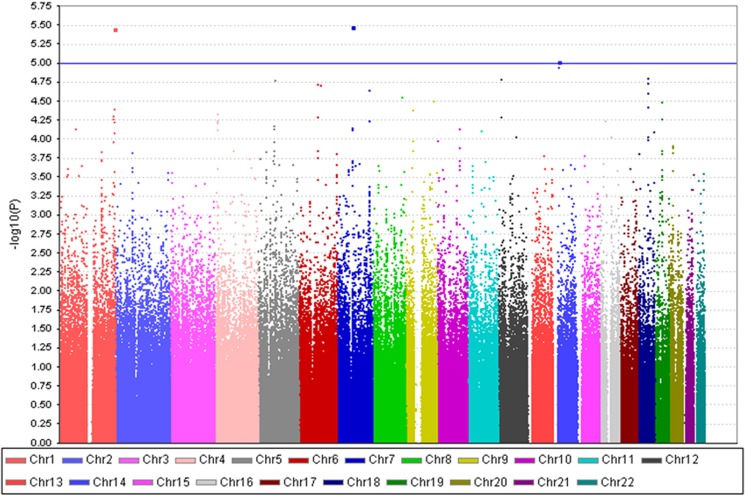
Manhattan plot of genome-wide association results in discovery phase sample. *P-*values are from the Cochran–Armitage trend test. For the replication phase, we selected candidate single-nucleotide polymorphisms (SNPs) whose *P*-value in the discovery phase was less than 1.0 × 10^−5^. The blue line indicates the cutoff probability value of replication (*P*=1.0 × 10^−5^). Two adjoined SNPs (rs7785360 and rs12698828) in chromosome 7 are represented as a single dot because of the short distance between them.

Four SNPs rs10924309, rs7785360, rs12698828 and rs8017553, all with *P*<1.0 × 10^−5^, were identified in the discovery set. These were then tested as candidate SNPs for the replication phase. These candidate SNPs resided on chromosomes 1q44, 7q11 and 14q11. The graphical summary of genome-wide association results is shown in a Manhattan plot (Figure 2). The results of association analyses for these four SNPs are shown in Table 2, and the top 100 ranked SNPs are presented in Supplementary Table S1. The detailed results including quality control, quantile–quantile plot (Supplementary Figure S1), and multidimensional scaling analysis (MDS plot, Supplementary Figure S2) are provided in Supplementary Results (Supplementary Text S1).

### Genetic-association analysis of SSRI treatment response in replication set

A focused replication study was performed for the four candidate SNPs from the three chromosomal loci shown in Table 2. Two intronic polymorphisms on 7q11.22 in perfect linkage disequilibrium (LD), rs7785360 and rs12698828, showed the most significant association with response in the replication set (nominal *P*=1.48 × 10^−3^, corrected *P*=0.006 Table 2). These two SNPs are part of the *AUTS2* gene in 7q11.22. The directions of association of these SNPs in the replication set were identical to those in the discovery set. None of the remaining two candidate SNPs was replicated (corrected *P*>0.05).

In a combined analysis of the discovery and replication sets, the two intronic SNPs (rs7785360 and rs12698828) of the *AUTS2* gene in 7q11.22 showed genome-wide significance (*P*=1.60 × 10^−8^) for association with antidepressant response to SSRIs. For each SNP, the minor allele frequency for responders in the combined set was lower than for non-responders (0.04 versus 0.11). In addition, these SNPs were not associated with any subject characteristics, drug choice or drop-out (*P*>0.05). This suggests that the associations of these two SNPs with response were not because of confounding factors.

**Figure 3:**
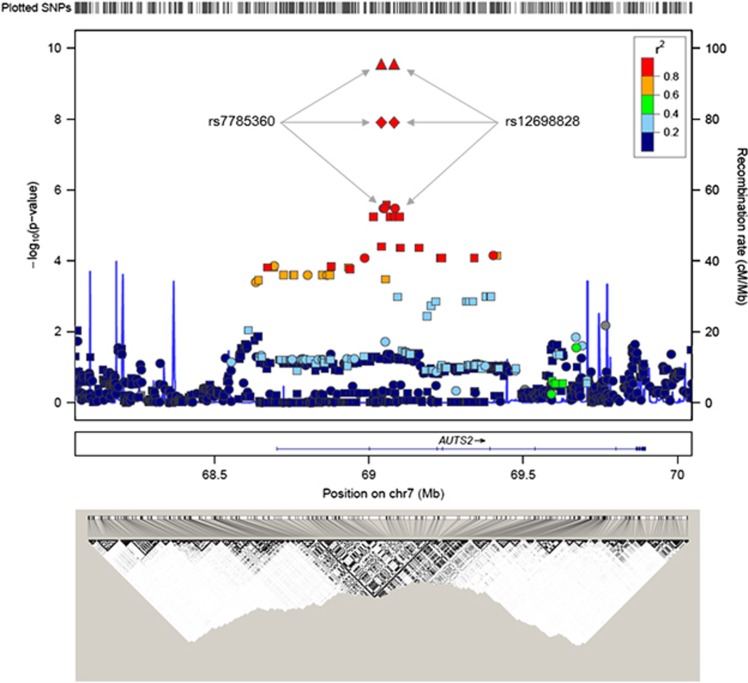
Regional association plot. *P-*values (ordinate axis, upper panel) are from the Cochran–Armitage trend test in the discovery set (circles: genotyped single-nucleotide polymorphisms (SNPs) and squares: imputed SNPs) and combination set (diamonds: the discovery and replication sets, triangles: discovery, replication and cross-replication set) in upper panel. The blue lines indicate the recombination rates in cM per Mb estimated using HapMap samples (upper panel). A horizontal line indicates the location of the *AUTS2* gene (middle panel). A linkage disequilibrium map based on *r*^2^ values was computed using the Hapmap JPT+CHB data (the International HapMap Project data, bottom panel).

Figure 3 shows regional associations centered at the chromosomal position of the replicated SNPs (upper panel). The significant SNPs are located in a LD block that has been identified in East Asian samples (Hapmap JPT+CHB data). The –log_10_(*P*) values abruptly dropped when they reached to the border of the LD block (lower panel).

### Genetic-association analysis of antidepressant response in cross-replication set

The two SNPs in the *AUTS2* gene (rs7785360 and rs12698828) on chromosome 7 that showed genome-wide significance in the combined discovery and replication sets were also associated with antidepressant response to mirtazapine in a focused study of the cross-replication set (nominal *P*=0.02, corrected *P*=0.04, Table 2). The directions of this association were identical to those in the SSRI-treated combined discovery and replication sets. For each SNP, the minor allele frequencies of responders were lower than for non-responders (0.04 versus 0.12). In addition, these SNPs were not associated with any subject characteristics, drug choice or drop-out in the cross-replication set (*P*>0.05).

### Genetic-association analysis of all-combined set

In a combined analysis for all analyzed completer patients (*n*=870), the two SNPs each showed genome-wide significance (*P*=6.60 × 10^−10^) for association with antidepressant response (Table 2) in univariate analysis. Next, we tested potential confounding variables of this association. Responders had fewer previous episodes, shorter duration of current episode, older age at onset and lower baseline HAM-D scores than non-responders in the all-combined set (Supplementary Table 2). In addition, number of previous episodes was significantly associated with the two SNPs (*P*=0.02), but gender, age, family history, duration of current episode, age at onset, baseline HAM-D score and drug choice were not. Therefore, number of previous episodes was considered as a possible confounding variable and entered in the multiple logistic regression model. The GWAS-level associations between each of these two SNPs and antidepressant response were preserved after controlling for gender, age, number of previous episodes, age at onset and baseline HAM-D scores (*P*=3.57 × 10^−8^, Supplementary Table 3). For each SNP, the response rate decreased (odds ratio=0.31, 95% confidence interval: 0.20–0.47) as a function of the number of minor alleles (non-response alleles). However, the explanatory power of this logistic regression model was relatively small (Cox and Snell's *R*^2^=0.09).^30^

### Genetic-association analysis of remission

The top 100 ranked SNPs in the discovery set for the association with remission are presented in Supplementary Table S4. The top results between response and remission were inconsistent. In addition, we tested the association between these SNPs and remission. The two SNPs in perfect LD did not show genome-wide significance (*P*=1.94 × 10^−4^) for association with the remission status in a combined analysis for all patients. This trend-level result was expected based on the smaller number of remitters compared with responders.

## Discussion

In this study we investigated the whole genomic associations of antidepressant response to SSRIs. We identified associations between response and two intronic SNPs (rs7785360 and rs12698828) in the *AUTS2* gene on 7q11.22, and we replicated these findings in an independent sample. In addition, we showed in a cross-replication set that these SNPs were also associated with antidepressant response to mirtazapine, a non-SSRI antidepressant, with identical directions to those in the SSRI-treated sets. In the combined discovery and replication samples of 711 patients who received SSRIs the identified SNPs achieved genome-wide significance (*P*=1.60 × 10^−8^). Likewise, genome-wide significance was determined for the combined three completer cohorts numbering 870 patients, with *P*=6.60 × 10^−10^ (Table 2). In addition, these significance levels were preserved after controlling for gender, age and number of previous episodes (*P*=2.35 × 10^−8^).

The *AUTS2* gene product is a nuclear protein that is expressed in the central nervous system in humans,^31^ especially in the cortical plate and ventricular zone, as well as the dentate gyrus, cornu ammonis (CA) 1 and CA3 areas of the hippocampus.^32^ The *AUTS2* gene has been repeatedly implicated in neurodevelopmental disorders including autism, intellectual disability and developmental delay.^32, 33, 34^ In studies of human evolution, the *AUTS2* gene was found to have significant changes between modern humans and Neanderthals, which has led to suggestions that *AUTS2* might be involved in cognitive traits specific to humans.^35, 36^ In addition, *AUTS2* expression has significant associations with nicotine dependence, cannabis dependence, alcohol sensitivity and antisocial personality.^32^ The *AUTS2* locus is implicated in schizoaffective or bipolar affective disorder patients.^32, 37, 38^ A recent pedigree analysis reported an association of the *AUTS2* gene and suicide.^39^ In the present study, we found two SNPs (rs7785360 and rs12698828) of the *AUTS2* gene associated with antidepressant response. This result was replicated and cross-replicated, as described in Results. A next-generation sequencing study^40^ in this locus will be helpful to identify functional variations and to clarify the function of this gene in neuropsychiatric disease.

In addition, we found that the genetic variations that showed significant associations with antidepressant response to SSRIs are also associated with response to another class of antidepressant drugs (mirtazapine). This result may suggest two possibilities. First, these SNPs (rs7785360 and rs12698828) may be linked with a final common action of different antidepressants. SSRIs increase the level of serotonin in the synaptic cleft. In addition, mirtazapine has a dual action profile on both the noradrenergic and serotonergic neurotransmitter systems.^41^ There is a possibility that the serotonergic system could be linked to an unknown pathway with which these SNPs are related. A previous protein expression study found that the *AUTS2* gene is expressed in frontocortical glutamatergic neurons.^42^ These excitatory neurotransmitter neurons are involved in mood circuitry.^43^ Depressed patients have abnormalities in glutamatergic neurotransmission.^44^ In addition, the glutamate system is modulated by antidepressants that affect serotonin.^45, 46^ Therefore, the glutamatergic system could be a connecting link between the *AUTS2* gene and antidepressant response. Another possible link is brain-derived neurotrophic factor. It has been shown that both SSRIs and mirtazapine induce brain-derived neurotrophic factor production in the brain.^47, 48^ In addition, brain-derived neurotrophic factor and the *AUTS2* gene have been linked to autism spectrum disorder.^49^ Further studies with the pathway of the *AUTS2* gene are required to clarify these possible connections. The second possibility is that these SNPs are associated with nonspecific improvement (placebo effect) rather than with specific antidepressant response. Similar to previous GWAS studies for antidepressant response,^7, 8, 9, 10^ our study was a naturalistic one; therefore, it did not have a placebo-treated group. Further studies with placebo-treated groups or groups treated with non-pharmacological interventions will be helpful to clarify this issue.

So far as we know, there have been no previous GWAS of antidepressant response among Asian patients except a Japanese pilot study with small sample size (*n*=92).^50^ In addition, this is the first GWAS that found significant associations between SNPs and response to multiple antidepressants in any ethnic group. Most previous GWAS studies were not able to identify associations that reached genome-wide statistical significance. Garriock *et al.*^7^ reported a GWAS of response to citalopram in patients of the Sequenced Treatment Alternatives to Relieve Depression (STAR*D) study. They suggested trend-level associations of three SNPs near the Ubiquitin protein ligase E3C (*UBE3C*) gene (rs6966038), the bone morphogenic protein 7 (*BMP7*) gene (rs6127921) and a third intronic SNP in the RAR-related orphan receptor alpha (*RORA*) gene (rs809736). Another group, the Munich Antidepressant Response Signature study, suggested different markers, also at a trend level: rs6989467 in the Cadherin-17 gene (*CDH17*) and rs1502174 in the Ephrin type-B gene (*EPHB1*).^8^ However, none of those suggested loci achieved GWAS significance. Another GWAS conducted by Uher *et al.*^9^ reported two gene markers with only 'suggestive' significance: rs2500535 in the interleukin-11 gene (*IL11*) and they reported that rs2500535 in the Uronyl 2-sulphotransferase gene (*UST*) showed significant association only in a subgroup analysis of patients who received nortriptyline. In addition, Tansey *et al.*^10^ reported negative results in a GWAS of over 2000 European-ancestry individuals with MDD. None of these associations was replicated, and the top results were also inconsistent among those studies, as well as with our study. None of our significant SNPs were replicated in a meta-analysis of the open data sets of STAR*D, GENDEP and Munich Antidepressant Response Signature (http://www.broadinstitute.org/mpg/ricopili/) that included only European-ancestry cases.^51^

We can suggest several reasons for such discrepancies in these GWAS studies of antidepressant response. The first is ethnicity: as Porcelli *et al.*^52^ reported, a genetic marker could have different significance according to the ethnicity of subjects. In a systematic meta-review of 33 studies, they showed that the serotonin transporter gene promoter polymorphism (*5-HTTLPR*) could be a predictor of antidepressant response and remission in Caucasians, whereas in Asians it does not appear to have a major role. Similarly, the STAR*D group showed that the association they observed between a gene (*HTR2A*) and treatment outcome of depression was confined to Caucasians.^53^ In addition, a GWAS of antidepressant response conducted by the STAR*D group reported no correlation of the SNP rankings across ancestry groups.^7^ These studies suggest a possibility that ethnic groups differ in their specific genetic markers for antidepressant response. Our previous report^54^ that investigated serotonin transporter genotype and function showed that functional differences associated with ethnicity could be a reason for this discrepancy of significant genetic markers. A recent investigation for the consistency of genome-wide associations across ancestral groups lends weight to this interpretation. Ntzani *et al.*^55^ analyzed more than a hundred genome-wide associations with nearly a thousand data sets, and reported differing risk estimates and considerable heterogeneity across ancestry groups. In addition, there is a possibility that the potential environmental modifiers could result in such discrepancies.^56^ In the field of antidepressant pharmacogenomics, further replication attempts, supported if possible by functional studies, in well-defined ethnic groups are needed.

Another possible factor for the lack of agreement between our results and previous reports is the age stratification of the depressed populations studied. Our patients were mostly of mature age (81.6% aged >50 years), and most of them (62.3%) had the first onset of depression after the age of 50 years. Previous studies that compared antidepressant response and age of first onset showed no relationship,^57, 58^ and some previous pharmacogenetic studies reported similar results with elderly and younger patients. However, several studies have suggested that age at onset can distinguish subtypes of depression, especially in terms of heritability.^59, 60^ Therefore, there is a possibility that the relatively large proportion of elderly patients in our study might be associated with reduced genetic and also clinical heterogeneity.

In a recent meta-analytic review, Undurraga and Baldessarini^61^ suggested that increasing subject numbers and recruitment sites have led to falling effect sizes in antidepressant trials. They recommended better quality control of diagnostic and clinical assessments as an alternative to the strategy of recruiting very large samples. From this point of view, strengths of our study include a limited number (2) of well-designed programs, managed by experienced research teams, with strictly blinded quality control. Other strengths of our study are ethnic homogeneity, confirmation of compliance by drug plasma level, inclusion of only clinically referred cases, clinical diagnoses by experienced psychiatrists in advance of confirmatory research diagnostic interviews^62^ and outcome assessments by consistent raters in person rather than by telephone. Moreover, we did not enroll subjects who had been exposed to any psychotropic drugs, including especially antidepressants, in the current episode of depression. By these means, heterogeneity and confounding of the case material were moderated in comparison with previous GWAS reports.

Although our sample is comparable in size to some previous GWAS reports,^8, 9, 11^ there are others with larger sample sizes that reported negative results.^7, 10^ However, we enrolled an ethnically homogeneous sample, as confirmed by the MDS plot (Supplementary Figure S2). In addition, the Korean population is genetically homogeneous.^63^ Therefore, we could avoid a population stratification step that might reduce statistical power in those previous studies, and we more precisely controlled the problem of ethnic heterogeneity than using rough classification by self-reported ancestry.

A potential limitation of our study is its naturalistic design. This design is the rule in GWAS of depression because it reflects the real world of depression management. With this design, clinical judgment enters into the choice of drug. Thus, there could be a selection bias for different drugs on the basis of prescriber preference and patient characteristics. However, any such selection bias would not affect the genetic associations with response in a drug class. A further limitation is that we found only a trend-level significance of association between the two SNPs and remission. This result was expected because the remission rate is always lower than the response rate in typical antidepressant trials;^29^ therefore, for adequate power a larger sample size would be required for analyses of remission. In addition, the low explanatory power of our result should be noted. It suggests that the prediction with the significant SNPs will not be sufficient for personalized treatment of depression at this stage. Another consideration is that because our patients were mostly elderly, the generalizability of our results to depressed patients in other age groups may be limited.

In conclusion, we conducted a GWAS of antidepressant drug response in Korean patients. We found a significant association between response to SSRIs and the two SNPs rs7785360 and rs12698828 in the *AUTS2* gene on chromosome 7, and the association generalized to response to mirtazapine with identical direction. These results may elucidate the common biological mechanisms of antidepressant drug action and they may further the search for genomic-based selection of antidepressant treatments. Further studies in a variety of ethnic populations will be required.

## Figures and Tables

**Table 1:** Clinical and demographic characteristics of analyzed completer cohorts

*Characteristics*	*Discovery set (*n=*481) SSRI-treated group*				*Replication set (*n=*230) SSRI-treated group*				P^a^	*Cross-replication set (*n=*159) mirtazapine-treated group*				P^b^
	*Total*	*Responder*	*Nonresponder*	P^c^	*Total*	*Responder*	*Nonresponder*	P^c^		*Total*	*Responder*	*Nonresponder*	P^c^	
Response rate	263 (54.7%)				118 (51.3%)				0.42	116 (73.0%)				<0.001
Remission rate	165 (34.3%)				79 (34.4%)				1	68 (42.8%)				0.06
Gender, female (%)^d^	354 (73.6%)	199 (75.7%)	155 (71.1%)	0.30	185 (80.4%)	103 (87.3%)	82 (73.2%)	0.01	0.05	115 (72.3%)	82 (70.7%)	33 (76.7%)	0.55	0.76
Age, year^e^	63 (54, 71)	64 (54, 70)	63 (54, 71)	0.09	60 (45, 68)	59.5 (44, 68)	60 (47, 68)	0.68	<0.0001	68 (60, 73)	67 (60, 74.5)	68 (62, 71)	0.28	<0.0001
Family history of depression (%)^d^	90 (18.7%)	46 (17.5%)	44 (20.2%)	0.48	46 (20.0%)	20 (17.0%)	26 (23.2%)	0.25	0.69	34 (21.4%)	25 (21.6%)	9 (20.9%)	1	0.49
Number of episodes^e^	2 (1, 2)	2 (1, 2)	2 (1, 3)	<0.01	2 (1, 3)	2 (1, 3)	2 (1, 3)	0.15	<0.01	2 (1, 3)	2 (1, 3)	3 (1, 5)	0.01	<0.0001
Duration of current episode, monthse,^f^	5 (2, 12)	4 (2, 9)	6 (3, 12)	<0.0001	5 (2, 12)	3 (2, 8)	6.5 (3, 16.5)	<0.0001	0.65	3 (2, 7)	3 (2, 8)	5 (2, 6)	0.91	0.03
Age at onset, year^e^	57 (45, 67)	58 (45, 67)	55 (43, 65)	0.12	50 (35, 62)	50 (39, 63)	49 (31, 59.5)	0.12	<0.0001	56 (48, 66)	56 (48, 66.5)	56 (42, 63)	0.25	0.53
HAM-D baseline^e^	19 (17, 23)	19 (17, 22)	20.5 (18, 24)	<0.0001	20 (18, 23)	20 (18, 23)	19 (17, 23)	0.05	0.15	18 (17, 21)	18 (17, 21)	20 (17, 22)	0.18	0.05
SSRIs														
Escitalopram	184 (38.3%)	94 (35.7%)	90 (41.3%)	0.54	148 (64.4%)	75 (63.6%)	73 (65.2%)	0.20						
Sertraline	110 (22.9%)	60 (22.8%)	50 (22.9%)		33 (14.4%)	15 (12.7%)	18 (16.1%)							
Fluoxetine	99 (20.6%)	56 (21.3%)	43 (19.7%)		25 (10.9%)	11 (9.3%)	14 (12.5%)							
Paroxetine	88 (18.3%)	53 (20.2%)	35 (16.1%)		24 (10.4%)	17 (14.4%)	7 (6.3%)							

Abbreviations: HAM-D, Hamilton depression rating score; SSRI, selective serotonin reuptake inhibitor.

a Comparison between discovery set and replication set, corrected by Bonferroni's correction for multiple testing.

b Comparison between discovery set and cross-replication set, corrected by Bonferroni's correction for multiple testing.

c Comparison between responders and non-responders.

d Fisher's exact test was used.

e Wilcoxon rank-sum test was used. Ranges shown are interquartile ranges.

f For duration of current episode *n*=481 in discovery set, *n*=145 in replication set and *n*=159 in cross-replication set. Data were not obtained for 85 patients of the Korea University Medical Center in replication set.

**Table 2:** The candidate SNPs associated with SSRI response (*P*<1.00 × 10^-5^) and their replication results

*SNP*	*Position*^a^	*Chromosome*	*Gene (±500 kb)*	*Cohort*	*Sample size*	P^b^	*Minor/major allele*	*MAF in responders*	*MAF in non-responders*
rs10924309	243929845	1q44	*KIF26B, SMYD3*	Discovery	481	3.51 × 10^-6^	A/G	0.53	0.38
				Replication	230	0.19		0.50	0.44
				Combined	711	6.01 × 10^-6^		0.52	0.40
rs7785360^c^	69047314	7q11	*AUTS2*	Discovery	481	3.28 × 10^-6^	T/C	0.03	0.11
				Replication	230	1.48 × 10^-3^		0.04	0.13
				Combined	711	1.60 × 10^-8^		0.04	0.11
				Cross-replication	159	0.02		0.04	0.12
				All-combined	870	6.60 × 10^-10^		0.04	0.11
rs12698828^c^	69084530			Discovery	481	3.28 × 10^-6^	C/G	0.03	0.11
				Replication	230	1.48 × 10^-3^		0.04	0.13
				Combined	711	1.60 × 10^-8^		0.04	0.11
				Cross-replication	159	0.02		0.04	0.12
				All-combined	870	6.60 × 10^-10^		0.04	0.11
rs8017553	22807362	14q11	*OXA1L, SLC7A7, MRPL52, MMP14, LRP10, REM2, RBM23, PRMT5, HAUS4, JUB, C14orf93, PSMB5, PSMB11, CDH24, SLC7A8, HOMEZ, PPP1R3E, BCL2L2, PABPN1, SLC22A17, EFS, CMTM5, MYH6, MIR208A, IL25,*	Discovery	481	9.45 × 10^-6^	C/T	0.27	0.15
				Replication	230	0.55		0.26	0.24
				Combined	711	1.21 × 10^-4^		0.26	0.18

Abbreviations: LD, linkage disequilibrium; MAF, minor allele frequency; SNP, single-nucleotide polymorphism; SSRI, selective serotonin reuptake inhibitor.

a Genomic position (NCBI Build 36).

b Nominal *P*-value of Cochran–Armitage trend test.

c These SNPs were in perfect linkage disequilibrium.
