# Supplementary material for: A genome-wide association study of antidepressant response in Koreans
Source: Transl Psychiatry. 2015 Sep 8;5(9):e633–. doi: 10.1038/tp.2015.127 (PMC5068817; doi:10.1038/tp.2015.127)
Supplement: Supplementary Information [file tp2015127x1.doc]

**Supplementary Online Methods**

***Flow of patients through the study***

Figure 1 shows the flow of patients through the study. Among the patients of Samsung Medical Center (SMC) who received a selective serotonin reuptake inhibitor (SSRI), 115 patients were excluded from analysis. Among them, 72 patients failed to attend scheduled clinic visits (31 escitalopram, 17 sertraline, 17 fluoxetine and 7 paroxetine), 30 patients discontinued treatment based on intolerable adverse effects (13 escitalopram, 9 sertraline, 4 paroxetine, and 4 fluoxetine) and 13 patients had undetectable plasma drug concentration, consistent with nonadherence (6 escitalopram, 3 fluoxetine, 2 paroxetine and 2 sertraline). A total of 645 patients (84.7%) completed the 6-week trial, and they were divided into discovery set (n=500) and replication set (n=145) based on the enrollment period. Following genotyping, an additional 19 subjects were excluded from the discovery set as a result of identity-by-descent analysis (IBD, see below). Thus, the final discovery set comprised 481 patients and the replication set comprised 145 patients. Among the patients of SMC who received mirtazapine, 12 patients who failed to attend scheduled clinic visits, 5 patients with undetectable plasma drug concentration, and 4 patients who discontinued treatment based on intolerable adverse effects were excluded from the cross-replication analysis. Finally, 159 patients comprised the cross-replication set.

An additional 99 patients for the replication set were recruited from the Pharmacogenomic Research Center for Psychotropic Drugs of the Department of Psychiatry, KUMC. All subjects were administered the SSRI drug escitalopram at a dose of 5-20mg/day. All patients were of unrelated Korean ancestry, and met the inclusion and exclusion criteria in force at the clinical trials program of the SMC.[^1^](#_ENREF_22) Their clinical symptoms were evaluated with HAM-D scales by a single trained rater at baseline and after 1, 2, 4 and 6 weeks of treatment. The response and remission criteria were the same as for SMC patients. Among the 99 patients of Korea University Medical Center (KUMC), 11 who did not follow scheduled clinic visits, and 3 patients who discontinued treatment based on intolerable adverse effects were excluded from analysis

***Plasma drug levels***

Plasma levels of antidepressant drugs were quantified through liquid or gas chromatography tandem mass spectrometry according to previously described methods in the patients from SMC.^2,3^

***SNP genotyping in discovery set***

We used the Affymetrix Genome-wide Human SNP array chip 6.0 for 905,431single nucleotide polymorphisms (SNPs) genotyping. Yields of pure, double-stranded genomic DNA were determined using the QIAamp DNA blood Maxi Kit (QIAGEN). Samples were normalized to 50ng/μL, and the normalized genomic DNA (5μL) from each sample was used as a template for Affymetrix Version 6.0 assays. Genotyping reactions were performed using Affymetrix Genome-Wide Human SNP Nsp/Sty, Version 6.0 kit reagents and protocols. Genotypes were called using the Birdseed v2 algorithm of Affymetrix Genotyping Console v3.0.2. The threshold of contrast quality control was 0.4, and the genotyping call rate was 95%. After genotyping, we performed identity-by-descent analysis to check the possible inclusion of related subjects, and 19-pairs of patients were suspected as related samples. (IBD>0.1875).^4^ We excluded the 19 patients of each pair who enrolled later in the discovery analysis. Then, we excluded SNPs which showed more than 1% missing rate or less than 5% minor allele frequency (MAF), *P-value* ≤0.001 by the result of Hardy-Weinberg equilibrium (HWE) test. We also excluded DNA samples which showed more than 5% missing rate. We selected candidate SNPs with p-value less than 1.0×10^-5^ as candidate SNPs for the replication set.

We also performed SNP imputation to increase the genome-wide coverage for further analyses. Imputation was performed by IMPUTE2 Version 2.3.0 ^5^ to impute 806,009 polymorphic SNPs that were not covered by the Affymetrix Version 6.0 array. We used the International HapMap Project data (phase 2 public Release #22 NCBI Build 36) for the reference panel. We confirmed that the position of common SNPs between the Affymetrix Version 6.0 array and the reference panel is identical. We pruned out poor imputed SNPs whose information value is less than 0.85. This information metric shows that 2 candidate SNPs, rs7785360 and rs12698828, were imputed with high certainty (IMPUTE2 information value = 1). We also used Haploview Version 4.1^6^ for assessing linkage disequilibrium structure. After imputation, we excluded samples that showed more than 20% missing rate. We also excluded SNPs that showed more than 1% missing rate and less than 5% MAF, p-value ≤ 0.001 by the HWE test.

***SNP genotyping in replication and cross-replication sets***

A total of 4 candidate SNPs (*P* < 1.00×10^-5^ in the discovery set) were genotyped in the replication and cross-replication sets using the MassARRAY system (Sequenom, San Diego, CA). Genotypes for SNPs that deviated from HWE in the replication set were confirmed by sequence analysis (Applied Biosystems), and SNPs of discordant genotypes were excluded from the analysis. We also excluded DNA samples and candidate SNPs that showed more than 5% missing rate.

***Power analysis***

The power of the discovery set (n=481) was calculated to be more than 89.6% for the Cochran-Armitage trend test under the significance level of 1.0×10^-5^. For the power calculation, we assumed that the response allele frequency was between 0.5 and 0.9, and that the probability of response increased by more than 0.25 when substituting one non-response allele with a response allele.

**Supplementary Online Results**

***Genetic association analysis of SSRI treatment response in discovery set***

No samples showed more than 5% missing rate. A total of 2,155 SNPs were excluded based on the HWE test. We removed 179,799 SNPs that showed more than 1% missing rate and 289,422 SNPs that showed less than 5% MAF. After filtering those SNPs, the association was tested between the SSRI response status and 505,435 SNPs. The distributions of observed *P-*values for association tests across all SNPs showed no evidence of overall systematic bias (λ = 1.00) from the expected *P-*values. We checked that confounders in the SSRI discovery set are not at work by using a quantile-quantile (QQ) plot (Supplementary Figure S1). In addition, we confirmed ethnic homogeneity by the multi-dimensional scaling analysis (MDS plot, Supplementary Figure S2).

**Supplementary Figure Legends**

**Supplementary Figure S1.** Quantile-quantile (QQ) plot for association test in the discovery set (inflation factor=1.00). No overall departures of the observed *P* values form the expected *P* values.

**Supplementary Figure S2.** Multi-dimensional scaling (MDS) plot of HapMap data and SSRI response discovery data. Each point represents an individual of different ethnicity; black circles indicate CEU (European ancestry), red triangles indicate JPT (Japanese ancestry) and CHB (Chinese ancestry), and green pluses indicate YRI (African ancestry) from the International HapMap Project data. Blue crosses indicate Korean discovery set patients. The x- and y-axis correspond to the first and second principal components derived by MDS.

**SUPPLEMENTARY REFERENCES**

1 Won ES, Chang HS, Lee HY, Ham BJ, Lee MS. Association between serotonin transporter-linked polymorphic region and escitalopram antidepressant treatment response in Korean patients with major depressive disorder. *Neuropsychobiology* 2012; **66**: 221-229.

2 Tournel G, Houdret N, Hedouin V, Deveaux M, Gosset D, Lhermitte M. High-performance liquid chromatographic method to screen and quantitate seven selective serotonin reuptake inhibitors in human serum. *Journal of Chromatography B: Biomedical Sciences and Applications* 2001; **761**: 147-158.

3. Maris F, Dingler E, Niehues S. High-performance liquid chromatographic assay with fluorescence detection for the routine monitoring of the antidepressant mirtazapine and its demethyl metabolite in human plasma. *Journal of Chromatography B: Biomedical Sciences and Applications* 1999; **721**: 309-316.

4 Anderson CA, Pettersson FH, Clarke GM, Cardon LR, Morris AP, Zondervan KT. Data quality control in genetic case-control association studies. *Nat Protoc* 2010; **5**: 1564-1573.

5 Howie BN, Donnelly P, Marchini J. A flexible and accurate genotype imputation method for the next generation of genome-wide association studies. *PLoS Genet* 2009; **5**: e1000529.

6 Barrett JC, Fry B, Maller J, Daly MJ. Haploview: analysis and visualization of LD and haplotype maps. *Bioinformatics* 2005; **21**: 263-265.
