# Supplementary material for: A genome-wide association study of antidepressant response in Koreans
Source: Transl Psychiatry. 2015 Sep 8;5(9):e633–. doi: 10.1038/tp.2015.127 (PMC5068817; doi:10.1038/tp.2015.127)
Supplement: Supplementary Table 1 [file tp2015127x2.doc]

**Table S1.** The top 100 ranked SNPs associated with response to SSRIs in discovery set

| **Rank** | **Chromosome** | **SNP** | **Minor Allele** | **Major Allele** | ***P*-value** |
| --- | --- | --- | --- | --- | --- |
| 1 | 7 | rs12698828 | C | G | 3.28E-06 |
| 2 | 7 | rs7785360 | T | C | 3.28E-06 |
| 3 | 1 | rs10924309 | A | G | 3.51E-06 |
| 4 | 14 | rs8017553 | C | T | 9.45E-06 |
| 5 | 14 | rs3811180 | T | C | 1.13E-05 |
| 6 | 18 | rs9635953 | G | A | 1.61E-05 |
| 7 | 12 | rs16912747 | G | A | 1.63E-05 |
| 8 | 5 | rs10515209 | G | C | 1.71E-05 |
| 9 | 18 | rs948667 | A | G | 1.85E-05 |
| 10 | 6 | rs11752615 | T | C | 1.90E-05 |
| 11 | 6 | rs1334328 | C | T | 1.98E-05 |
| 12 | 7 | rs2091104 | C | T | 2.27E-05 |
| 13 | 18 | rs7244563 | T | C | 2.50E-05 |
| 14 | 8 | rs4332094 | C | T | 2.79E-05 |
| 15 | 9 | rs10985450 | G | C | 3.14E-05 |
| 16 | 19 | rs877836 | T | C | 3.29E-05 |
| 17 | 18 | rs2276170 | A | G | 3.81E-05 |
| 18 | 1 | rs10924305 | A | G | 4.09E-05 |
| 19 | 9 | rs12353109 | C | T | 4.21E-05 |
| 20 | 4 | rs1520313 | G | A | 4.64E-05 |
| 21 | 1 | rs2165872 | T | C | 4.98E-05 |
| 22 | 6 | rs11754643 | A | G | 5.14E-05 |
| 23 | 12 | rs10772898 | T | C | 5.17E-05 |
| 24 | 1 | rs1867264 | T | A | 5.38E-05 |
| 25 | 1 | rs1867265 | T | C | 5.38E-05 |
| 26 | 19 | rs11669149 | G | A | 5.46E-05 |
| 27 | 4 | rs17366799 | A | G | 5.83E-05 |
| 28 | 7 | rs12669573 | C | T | 5.83E-05 |
| 29 | 16 | rs3826263 | G | C | 5.86E-05 |
| 30 | 1 | rs6700721 | T | C | 5.91E-05 |
| 31 | 4 | rs1104703 | T | A | 6.11E-05 |
| 32 | 5 | rs6453086 | A | G | 6.77E-05 |
| 33 | 23 | rs984230 | A | G | 6.81E-05 |
| 34 | 7 | rs12698891 | C | T | 7.19E-05 |
| 35 | 10 | rs2297988 | G | A | 7.28E-05 |
| 36 | 5 | rs2044410 | C | T | 7.37E-05 |
| 37 | 1 | rs2114213 | G | T | 7.43E-05 |
| 38 | 7 | rs12698811 | G | A | 7.61E-05 |
| 39 | 4 | rs1402038 | A | G | 7.62E-05 |
| 40 | 11 | rs3741255 | A | G | 7.87E-05 |
| 41 | 18 | rs750123 | G | A | 7.98E-05 |
| 42 | 1 | rs6701023 | C | G | 8.34E-05 |
| 43 | 16 | rs4785465 | T | G | 9.34E-05 |
| 44 | 12 | rs2263942 | G | T | 9.37E-05 |
| 45 | 18 | rs7244073 | A | G | 9.37E-05 |
| 46 | 18 | rs7244102 | A | G | 1.03E-04 |
| 47 | 23 | rs6610384 | C | T | 1.05E-04 |
| 48 | 10 | rs11252385 | C | T | 1.07E-04 |
| 49 | 9 | rs10123547 | T | G | 1.07E-04 |
| 50 | 1 | rs1867263 | T | C | 1.09E-04 |
| 51 | 5 | rs1993370 | T | C | 1.10E-04 |
| 52 | 20 | rs6136530 | C | T | 1.22E-04 |
| 53 | 20 | rs6136525 | G | A | 1.29E-04 |
| 54 | 10 | rs10882907 | C | G | 1.29E-04 |
| 55 | 6 | rs13212099 | A | G | 1.41E-04 |
| 56 | 19 | rs1030100 | G | A | 1.42E-04 |
| 57 | 9 | rs10970922 | A | G | 1.42E-04 |
| 58 | 4 | rs676592 | C | T | 1.42E-04 |
| 59 | 5 | rs10062244 | T | C | 1.42E-04 |
| 60 | 5 | rs10491377 | C | T | 1.47E-04 |
| 61 | 1 | rs12046366 | C | A | 1.48E-04 |
| 62 | 1 | rs7555408 | C | T | 1.48E-04 |
| 63 | 20 | rs2328452 | T | C | 1.52E-04 |
| 64 | 2 | rs6747145 | T | C | 1.54E-04 |
| 65 | 2 | rs7576824 | C | T | 1.54E-04 |
| 66 | 19 | rs1024881 | C | G | 1.54E-04 |
| 67 | 18 | rs11663529 | A | G | 1.55E-04 |
| 68 | 6 | rs3800544 | T | C | 1.56E-04 |
| 69 | 5 | rs17738645 | G | C | 1.63E-04 |
| 70 | 13 | rs9564791 | T | C | 1.65E-04 |
| 71 | 15 | rs6495009 | A | C | 1.68E-04 |
| 72 | 15 | rs7173234 | C | T | 1.68E-04 |
| 73 | 6 | rs11756746 | A | G | 1.74E-04 |
| 74 | 4 | rs6818398 | C | T | 1.79E-04 |
| 75 | 5 | rs12519432 | G | A | 1.80E-04 |
| 76 | 1 | rs1912911 | C | A | 1.85E-04 |
| 77 | 1 | rs4388707 | T | C | 1.91E-04 |
| 78 | 5 | rs4700160 | C | T | 1.91E-04 |
| 79 | 10 | rs4635018 | C | G | 1.92E-04 |
| 80 | 7 | rs1003404 | T | C | 1.93E-04 |
| 81 | 1 | rs2861430 | C | A | 1.93E-04 |
| 82 | 1 | rs6428096 | A | G | 1.93E-04 |
| 83 | 11 | rs7947523 | C | G | 1.98E-04 |
| 84 | 16 | rs17205838 | G | C | 2.09E-04 |
| 85 | 5 | rs17738111 | C | T | 2.09E-04 |
| 86 | 5 | rs17556534 | G | C | 2.18E-04 |
| 87 | 5 | rs258952 | G | A | 2.20E-04 |
| 88 | 14 | rs1004667 | A | G | 2.20E-04 |
| 89 | 6 | rs6939896 | T | C | 2.20E-04 |
| 90 | 7 | rs4464906 | G | A | 2.22E-04 |
| 91 | 11 | rs7109421 | A | G | 2.22E-04 |
| 92 | 10 | rs7092350 | T | A | 2.23E-04 |
| 93 | 10 | rs7092499 | G | A | 2.23E-04 |
| 94 | 10 | rs9888026 | G | T | 2.23E-04 |
| 95 | 10 | rs9888117 | G | C | 2.23E-04 |
| 96 | 1 | rs12565150 | A | T | 2.24E-04 |
| 97 | 8 | rs1425725 | A | G | 2.24E-04 |
| 98 | 15 | rs10775205 | T | C | 2.25E-04 |
| 99 | 7 | rs1990444 | T | C | 2.31E-04 |
| 100 | 4 | rs1373469 | A | G | 2.35E-04 |
