# Supplementary material for: A genome-wide association study of antidepressant response in Koreans
Source: Transl Psychiatry. 2015 Sep 8;5(9):e633–. doi: 10.1038/tp.2015.127 (PMC5068817; doi:10.1038/tp.2015.127)
Supplement: Supplementary Table 2 [file tp2015127x3.doc]

**Supplementary Table S2. Clinical and Demographic Characteristics of all-combined set (n=870)**

| **Characteristics** | **Total** | **Responder (n=497)** | **Nonresponder (n=373)** | ***P*** |
| --- | --- | --- | --- | --- |
| Gender, Female (%) ^a^. | 654 (75.2%) | 384 (77.3%) | 270 (72.4%) | 0.11 |
| Age, Year ^b^. | 63 (53, 71) | 64 (54, 71) | 63 (53, 70) | 0.13 |
| Family History of Depression (%) ^a^. | 170 (19.5%) | 91 (18.3%) | 79 (21.2%) | 0.30 |
| Number of Episodes ^b^. | 2 (1,3) | 2 (1,2) | 2 (1,3) | <0.001 |
| Duration of current episode, Months ^b. c^. | 4 (2, 11) | 3 (2,9) | 6 (3, 12) | <0.0001 |
| Age at Onset, Year ^b^. | 55 (42, 65) | 56 (43, 67) | 53 (39, 63) | <0.01 |
| HAM-D Baseline ^b^. | 19 (17, 22) | 18 (17, 22) | 20 (18, 23) | <0.0001 |

Abbreviation: HAM-D, Hamilton depression rating scale; SSRIs: Selective Serotonin Reuptake Inhibitors

^a^. Fisher’s exact test was used

^b^. Wilcoxon rank sum test was used. Ranges shown are Inter-Quartile Ranges.

^c^. For duration of current episode n=785, with 448 responders and 337 nonresponders (see legend to Table 1). Data for this variable were not obtained for 85 patients of Korea University Medical Center in replication set.
