# Supplementary material for: A genome-wide association study of antidepressant response in Koreans
Source: Transl Psychiatry. 2015 Sep 8;5(9):e633–. doi: 10.1038/tp.2015.127 (PMC5068817; doi:10.1038/tp.2015.127)
Supplement: Supplementary Table 3 [file tp2015127x4.doc]

**Supplementary Table S3. Logistic regression analysis for antidepressant response in all-combined set (n=870)**

| **Variables** | **Regression Coefficient** | **Standard Error** | **OR (95% CI)** | ***P*** |
| --- | --- | --- | --- | --- |
| Gender (Female) | 0.32 | 0.17 | 1.38 (1.00–1.91) | 0.05 |
| Age, Year | 0.01 | 0.01 | 1.01 (0.99–1.03) | 0.49 |
| Number of Episodes | -0.19 | 0.06 | 0.83 (0.74–0.92) | 8.02×10^-4^ |
| Age at onset | -0.002 | 0.01 | 1.00 (0.98–1.02) | 0.82 |
| Baseline HAM-D | -0.08 | 0.02 | 0.93 (0.89–0.96) | 3.40×10^-5^ |
| Number of minor alleles | -1.17 | 0.21 | 0.31 (0.20–0.47) | 3.57×10^-8^ |

Abbreviation: OR, Odds ratio; CI, Confidence Interval.

These analyses were conducted for each significant SNP (rs7785360 and rs12698828) in perfect linkage disequilibrium (LD).
