# Supplementary material for: A genome-wide association study of antidepressant response in Koreans
Source: Transl Psychiatry. 2015 Sep 8;5(9):e633–. doi: 10.1038/tp.2015.127 (PMC5068817; doi:10.1038/tp.2015.127)
Supplement: Supplementary Table 4 [file tp2015127x5.doc]

**Table S4.** The top 100 ranked SNPs associated with remission to SSRIs in discovery set

| **Rank** | **Chromosome** | **SNP** | **Minor Allele** | **Major Allele** | ***P*-value** |
| --- | --- | --- | --- | --- | --- |
| 1 | 7 | rs4632976 | A | C | 2.18E-06 |
| 2 | 7 | rs2372053 | A | C | 2.98E-06 |
| 3 | 7 | rs7457799 | C | T | 3.58E-06 |
| 4 | 4 | rs12510934 | C | T | 7.60E-06 |
| 5 | 7 | rs6464681 | T | C | 8.13E-06 |
| 6 | 15 | rs7172133 | C | A | 1.82E-05 |
| 7 | 1 | rs7545677 | C | T | 1.90E-05 |
| 8 | 7 | rs1229569 | T | C | 1.99E-05 |
| 9 | 8 | rs2385675 | C | T | 2.51E-05 |
| 10 | 5 | rs2061714 | T | C | 2.81E-05 |
| 11 | 13 | rs667374 | T | C | 3.02E-05 |
| 12 | 12 | rs704205 | G | T | 3.15E-05 |
| 13 | 1 | rs12068202 | A | G | 3.20E-05 |
| 14 | 23 | rs5951582 | T | C | 3.52E-05 |
| 15 | 8 | rs7826788 | A | G | 3.85E-05 |
| 16 | 1 | rs12731740 | T | C | 4.50E-05 |
| 17 | 9 | rs12353109 | C | T | 4.70E-05 |
| 18 | 5 | rs6864917 | C | T | 4.80E-05 |
| 19 | 1 | rs4845200 | G | A | 4.89E-05 |
| 20 | 5 | rs11745874 | T | C | 4.93E-05 |
| 21 | 10 | rs1574317 | A | G | 5.24E-05 |
| 22 | 8 | rs1376013 | T | C | 5.34E-05 |
| 23 | 9 | rs10116558 | T | C | 5.58E-05 |
| 24 | 1 | rs2944677 | T | A | 5.96E-05 |
| 25 | 7 | rs850499 | T | C | 5.96E-05 |
| 26 | 1 | rs11121090 | A | G | 5.96E-05 |
| 27 | 8 | rs6992419 | T | G | 6.00E-05 |
| 28 | 10 | rs1574318 | A | G | 6.21E-05 |
| 29 | 23 | rs5917358 | C | G | 6.25E-05 |
| 30 | 19 | rs10422843 | A | C | 6.33E-05 |
| 31 | 1 | rs11208013 | G | T | 6.42E-05 |
| 32 | 1 | rs6703955 | G | C | 6.42E-05 |
| 33 | 1 | rs4908727 | C | T | 6.47E-05 |
| 34 | 4 | rs4689007 | A | G | 6.48E-05 |
| 35 | 6 | rs9367529 | G | T | 6.48E-05 |
| 36 | 8 | rs7463321 | T | C | 6.91E-05 |
| 37 | 1 | rs7538847 | T | C | 7.27E-05 |
| 38 | 4 | rs1104704 | C | T | 7.27E-05 |
| 39 | 6 | rs9367389 | G | A | 7.49E-05 |
| 40 | 7 | rs6952652 | T | C | 7.54E-05 |
| 41 | 1 | rs12062955 | T | G | 7.67E-05 |
| 42 | 1 | rs1568132 | A | G | 7.67E-05 |
| 43 | 1 | rs1581231 | C | G | 7.67E-05 |
| 44 | 1 | rs2138673 | C | T | 7.67E-05 |
| 45 | 1 | rs3005684 | C | T | 7.67E-05 |
| 46 | 1 | rs3005705 | T | C | 7.67E-05 |
| 47 | 8 | rs16906934 | C | T | 7.69E-05 |
| 48 | 9 | rs10869034 | G | T | 7.75E-05 |
| 49 | 20 | rs1883269 | C | T | 7.83E-05 |
| 50 | 4 | rs6532120 | A | G | 8.03E-05 |
| 51 | 9 | rs10123547 | T | G | 8.04E-05 |
| 52 | 1 | rs2944678 | G | C | 8.13E-05 |
| 53 | 7 | rs850502 | G | A | 8.45E-05 |
| 54 | 8 | rs11987235 | G | A | 9.11E-05 |
| 55 | 8 | rs16906925 | T | G | 9.16E-05 |
| 56 | 23 | rs7054029 | A | G | 9.43E-05 |
| 57 | 6 | rs12207943 | G | A | 9.67E-05 |
| 58 | 6 | rs12528705 | G | A | 9.67E-05 |
| 59 | 6 | rs1340856 | C | A | 9.67E-05 |
| 60 | 6 | rs1417503 | A | G | 9.67E-05 |
| 61 | 6 | rs1417506 | C | T | 9.67E-05 |
| 62 | 6 | rs6906643 | G | T | 9.67E-05 |
| 63 | 6 | rs7769175 | T | A | 9.67E-05 |
| 64 | 6 | rs9369942 | C | T | 9.67E-05 |
| 65 | 6 | rs9395562 | A | G | 9.67E-05 |
| 66 | 7 | rs1976135 | C | G | 0.0001 |
| 67 | 22 | rs470094 | A | G | 0.000103 |
| 68 | 7 | rs12374872 | G | A | 0.000104 |
| 69 | 8 | rs9650068 | G | A | 0.000104 |
| 70 | 7 | rs1976132 | C | G | 0.000106 |
| 71 | 13 | rs634035 | C | T | 0.000108 |
| 72 | 1 | rs10495081 | A | G | 0.000111 |
| 73 | 7 | rs16882782 | C | T | 0.000114 |
| 74 | 9 | rs10970922 | A | G | 0.000114 |
| 75 | 6 | rs11755082 | A | C | 0.000115 |
| 76 | 1 | rs12030969 | T | C | 0.000115 |
| 77 | 7 | rs16873051 | C | G | 0.000117 |
| 78 | 11 | rs12364612 | C | T | 0.000119 |
| 79 | 11 | rs12419719 | C | T | 0.000119 |
| 80 | 4 | rs17147943 | A | T | 0.00012 |
| 81 | 6 | rs1417507 | A | G | 0.000122 |
| 82 | 6 | rs6910805 | T | C | 0.000122 |
| 83 | 6 | rs4715179 | A | G | 0.000122 |
| 84 | 9 | rs17588480 | T | C | 0.000124 |
| 85 | 9 | rs3920702 | C | T | 0.000124 |
| 86 | 15 | rs11073911 | C | T | 0.000124 |
| 87 | 1 | rs1517105 | C | T | 0.000125 |
| 88 | 7 | rs10253538 | G | A | 0.000125 |
| 89 | 1 | rs6676845 | T | C | 0.000127 |
| 90 | 8 | rs41535046 | A | G | 0.000129 |
| 91 | 17 | rs8072687 | A | G | 0.000132 |
| 92 | 17 | rs9916638 | C | T | 0.000132 |
| 93 | 6 | rs6921517 | A | G | 0.000132 |
| 94 | 4 | rs1603931 | G | A | 0.000132 |
| 95 | 9 | rs1411992 | G | C | 0.000134 |
| 96 | 14 | rs8017553 | C | T | 0.00014 |
| 97 | 6 | rs9378305 | A | G | 0.00014 |
| 98 | 7 | rs17154033 | C | G | 0.000141 |
| 99 | 6 | rs987475 | C | A | 0.000144 |
| 100 | 9 | rs6560203 | G | A | 0.000147 |
