# Supplementary figures and images for: A genome-wide association study of antidepressant response in Koreans
Source: Transl Psychiatry. 2015 Sep 8;5(9):e633–. doi: 10.1038/tp.2015.127 (PMC5068817; doi:10.1038/tp.2015.127)

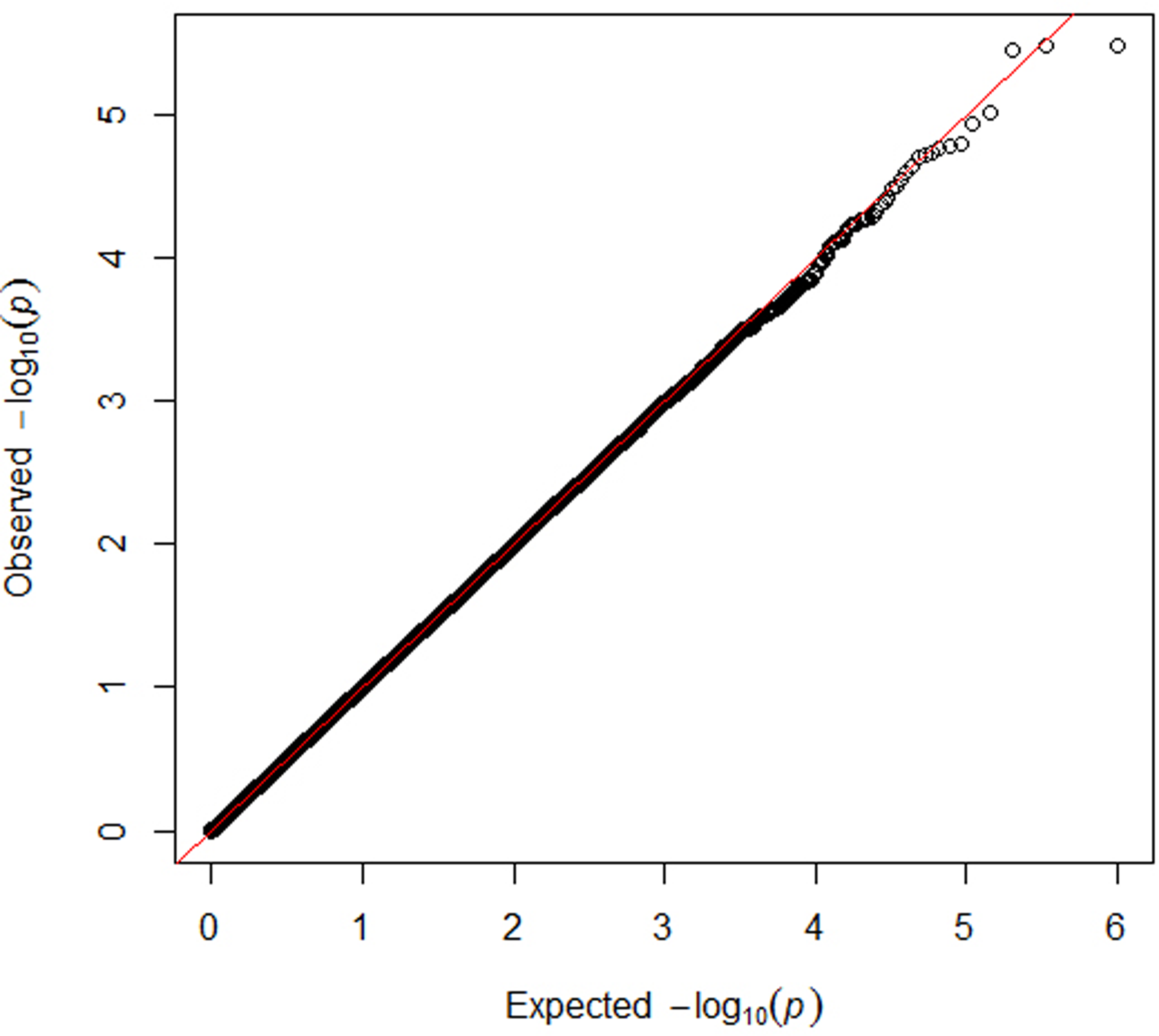

Supplement: Supplementary Figure 1 [file tp2015127x6.tif]

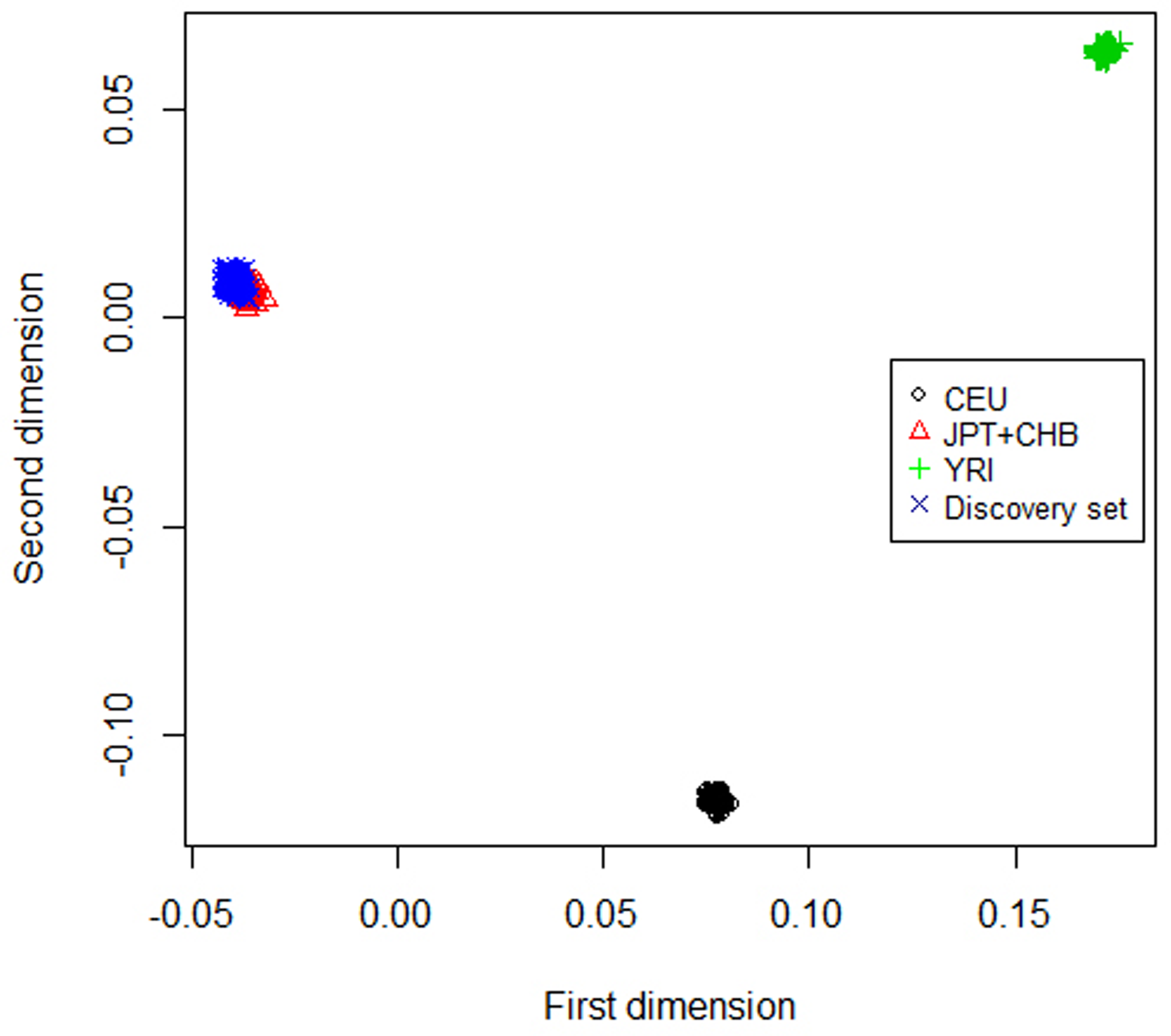

Supplement: Supplementary Figure 2 [file tp2015127x7.tif]
